# Pure tubular carcinoma of the breast: a case series

**DOI:** 10.1186/s13256-023-03950-w

**Published:** 2023-06-27

**Authors:** S. Sakhri, M. Aloui, I. Zemni, S. Kamoun, M. Slimene, K. Rahal

**Affiliations:** 1 Surgical Oncology Department, Salah Azaiez Institute of Oncology, Tunis, Tunisia; 2 Pathology Department, Salah Azaiez Institute of Oncology, Tunis, Tunisia

**Keywords:** Pure tubular breast carcinoma, Radiology, Prognosis, Surgery, Adjuvant treatment

## Abstract

**Background:**

Pure tubular breast carcinoma is a rare and well-differentiated tumor with high survival and low local recurrence rate. Our study aims to determine the clinical, radiological, appropriate management, and prognosis of this carcinoma.

**Materials and methods:**

A review of Salah Azaiez institute registry from 2004 to 2019 was performed including seven cases of PTC of the breast.

**Results:**

Clinical-pathologic features and outcomes were analyzed. The median follow-up was 3 years. In our study, we found that the cohort presented more frequently with pT1 disease and pN0 disease. Conservative surgery was more frequently indicated (five cases). All patients had hormone-receptor positivity and Human Epidermal growth factor Receptor 2 (*HER2*) negativity. The majority of tumors had a molecular profile luminal A and a low-grade SBR. In one case we found axillary lymph node metastasis. Adjuvant radiotherapy was indicated in all cases of breast conservation and in only one case of radical surgery. One patient received chemotherapy. The mean follow-up was 4 years. We did not find any local or distant recurrence in our study.

**Conclusion:**

PTC showed an excellent prognosis with a low SBR grade, a molecular profile luminal A, and a low incidence of recurrence.

## Introduction

Pure tubular carcinoma (PTC) of the breast was first described by Cornil and Ranvier in 1869 as a distinct entity [1]. It is an uncommon subtype of invasive breast cancer accounting for less than 2% of invasive breast cancers and about 1% of all breast carcinomas. It is characterized by well-differentiated and regular cells arranged in well-defined tubules mimicking breast ductules. This tumor is nearly always estrogen (ER) and progesterone receptor (PR)-positive, and mostly human epidermal growth factor receptor type 2 (*HER2*)-negative [2].

PTC typically occurs in women over 50. But it is very rare in men. It is associated with an excellent prognosis [3].

## Methods

We conducted a retrospective study at Salah Azaiez Institute of oncology. All patients diagnosed with pure tubular carcinoma of the breast from January 2004 to March 2019 were gathered, giving a total of seven patients. Information were collected for each patient with respect to medical history, mode of detection of lesions (on a screening mammogram or a palpable mass), tumor pathology, treatment received, and patient follow-up. Further details regarding each patient’s history were taken into consideration including age, gender, menopausal status, personal history of breast or other cancer, and family history of breast cancer. The characteristics of each cancer were tracked, including TNM staging, pathologic subtype, and hormonal status. Treatments were categorized as the type of surgery, the type of adjuvant therapy, if any, and whether radiation was included in the treatment. Types of surgery included breast conservation therapy, total mastectomy with sentinel lymph node biopsy, and radical mastectomy. Patient follow-up documentation provided recurrence and survival data.

## Results

A total of seven patients were included in this study. The average age was 54 years old (from 36 to 70 years) and all patients were female. One of the patients had a personal history of breast cancer. None of them had a family history of breast cancer. Five women were in peri- or postmenopausal. The diagnosis was made by mammography in two cases. Six cancers were clinically detected on physical examination.

The majority of patients in this series were diagnosed with early-stage breast cancer. In regards to tumor size (T stage), three cases were T1, two were T2, and the remaining two were T2m. A clinical lymph node was found in only one case.

The mammography was free of lesions in three cases, showed a benign lesion in three cases, and a suspicious lesion in one case. Microcalcifications appeared in two cases. The ultrasonography showed a benign hypoechogenic lesion in all cases. The median size of tumors was 10 mm. The radiological assessment did not show any metastasis. Three patients had a negative biopsy, two had a benign lesion, and one biopsy showed an invasive ductal carcinoma.

Breast conservation therapy was performed in five cases, and two patients underwent a mastectomy. All patients underwent axillary lymph node staging. Only four cases underwent sentinel lymph node biopsy, and the other patients underwent lymph node dissection.

In the histological examination, all the patients were diagnosed with pure tubular carcinoma; the average size of the tumor was 10 mm. It was multifocal in two cases, and in all cases, the tumor was SBR I. In this series, only one patient had axillary lymph node metastasis.

In regards to tumor biology, all patients were both ER and PR positive, and negative for *HER2*/neu. Only one patient had a profile luminal B; the other patients had a profile luminal A.

All patients who underwent conservative breast surgery were offered adjuvant breast radiation therapy. Among those who underwent radical surgery, only one patient received adjuvant radiotherapy associated with chemotherapy based on 3 epirubicin, cisplatin, 5-fluorouracil (EFC) + 3 docetaxel (TXT). In only one of the cases was adjuvant radiation not received (because of the difficulty in access to healthcare during the Tunisian revolution).

All patients with hormone-positive cancers were offered endocrine treatment (tamoxifen or aromatase inhibitor) for 5 years.

The mean follow-up was 4 years. None of the patients developed local recurrence, even in the case of the patient who did not receive adjuvant radiotherapy. There were no cancer-related mortalities.

## Discussion

Tubular carcinoma is classified as pure or mixed. According to the new World Health Organization (WHO) classification, tubular carcinoma is called pure when more than 90% of the tumor exhibits the tubular growth pattern with a low nuclear grade and no mitoses. Some studies have suggested that low-grade ductal carcinoma *in situ* (DCIS) can be a precursor for tubular breast carcinoma by documenting the association between tubular carcinoma and micropapillary and cribriform DCIS [4].

PTC is more frequent in populations where there is screening mammography, which is why it is predominant in women in their sixth decade who underwent breast cancer screening [5]. This carcinoma is often first detected on screening mammography because it is usually very small (< 1 cm) and not palpable and it is classified as T1. In the literature, the average size of PTC was 0.8 cm in patients with nonpalpable lesions, and 1.2 cm when the lesions were palpable [1]. These results are similar to our findings, as the majority of our patients were diagnosed with early-stage breast cancer. The increase in frequency is due to the development of the screening. In fact, in Tunisia, we do not have an organized screening program. However, the Ministry of Health is organizing screening campaigns for women aged 40 and over by mammography. For young patients with high risk, an MRI is indicated from the age of 20 years. In regards to tumor size (T stage), three cases were T1. The tumor is localized more frequently in the upper outer quadrant of the breast. The multifocality was reported in 20% of the cases [7].

Radiologically, some cases of PTC are difficult to identify on traditional mammography, especially in dense breasts. In this case, breast ultrasound can help to detect the tumor. But in the majority of cases, the mammography was abnormal (80%). This carcinoma typically appears as a small mass that may appear round, oval, or lobulated, with irregular or spiculated margins. The spicules are often longer than the central mass. It can be associated with suspicious microcalcifications in 8–9% of cases [1]. In some cases, it can appear as an architectural distortion. In the majority of cases, the appearance mimics typical intraductal carcinoma (IDC) [10]. Ultrasonography is helpful to detect occult lesions in mammography; PTC presents as a hypoechoic solid mass with ill-defined margins and posterior acoustic shadowing mimicking IDC [10]. MRI imaging is performed rarely in PTC because of the typical malignant aspect on mammography and ultrasound, and because of the small size, the PTC presented as an irregular mass with a type 3 enhancement curve. It is helpful in cases where lesions are only seen on one mammographic view [3].

In the current study, the mammography was free of lesions in three cases, showed a benign lesion in three cases, and a suspicious lesion in one case. Microcalcifications appeared in two cases. The ultrasonography showed a benign hypoechogenic lesion in all cases.

Surgery is usually the first treatment for PTC, and can be conserving surgery or mastectomy; in this case, the patient can benefit from an immediate reconstruction or delayed reconstruction [1]. Because of the small size (less than 1 cm) of the tumor, breast-conserving surgery is the main local treatment for PTC. It consists of a lumpectomy and surgical axillary node staging [8].

However, the need for axillary staging in these patients is questionable. Some authors suggested that axillary staging may be unnecessary in patients with small tumors (less than 1 cm), since the tumor size is considered the main risk for positive axillary lymph nodes, and because of the excellent survival rate of this type of carcinoma and seeing as it is less likely to spread to the lymph nodes [4]. In the present study, most of the patients underwent conservative surgery and all of them underwent an axillary lymph node staging. Lymph node sentinel biopsy was done in five cases, in the other two cases the patients underwent a total lymph node dissection.

However, currently, there is no consensus on the omission of surgical axillary node staging [4]. Contrariwise, since the incidence of lymph node metastases may range from 4% to 17% [7], it is necessary to do a sentinel lymph node biopsy (SLNB) to check if axillary lymph nodes have been affected, because the positive biopsy will indicate whether adjuvant treatment is needed [7].

Kara *et al.* and Jung *et al. *concluded that the use of axillary staging for patients with PCT is necessary because they found that the incidence of axillary lymph node metastasis was 18% in their study [10]. In our study, only one patient had a metastatic lymph node (1N^+^/17 nodes).

Currently, recent studies think that the survival of patients with PTC is similar to the general population, so there is no evidence that adjuvant therapy due to positive SLNB influences survival [9]. But there is no clear recommendation for the omission of the use of axillary staging.

**Fig. 1:**
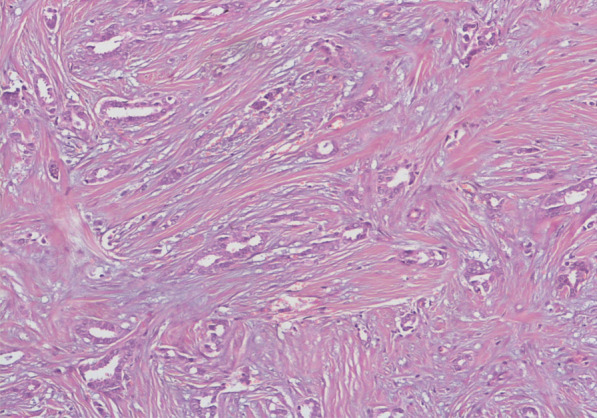
Hematoxylin and eosin (HE) × 100: well-differentiated tubules surrounded by an abundant fibrous stroma

In histopathology, PTC often presents as a white and ill-defined limit, firm or hard mass measuring between 0.2 cm and 2 cm in diameter [7]. Tubular carcinoma is composed of well-differentiated tubules with open lumina, typically surrounded by abundant stroma (Fig. 1). It may contain other histologic elements. An excess of 90% of tubular elements are usually required for the diagnosis of PTC. The gland usually lacks myoepithelial cells, and the lesions can be multifocal or multicentric [3, 7].

**Fig. 2:**
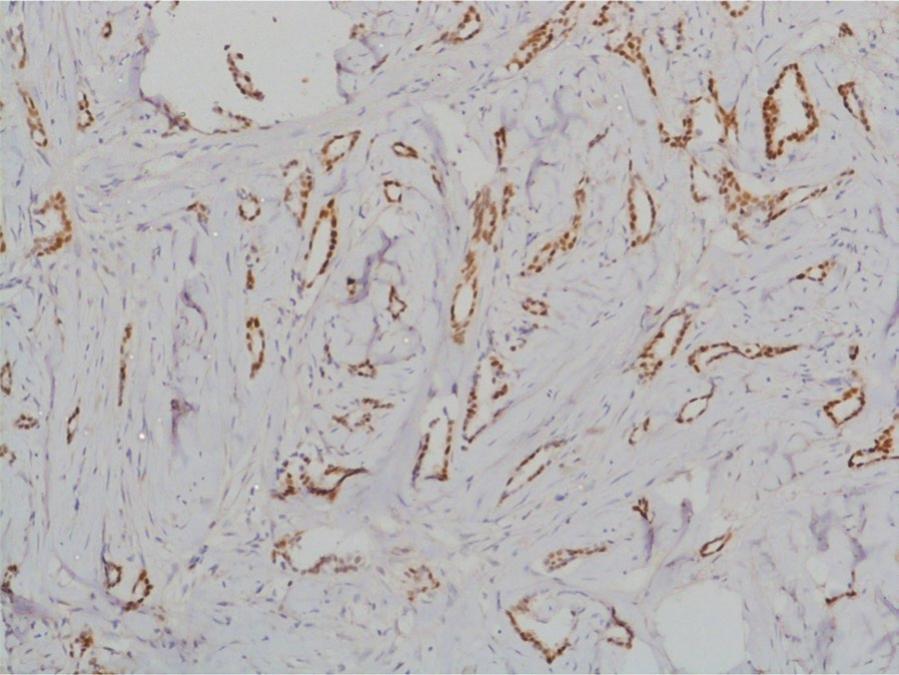
IHC × 100 RE showing positive and diffuse staining for estrogen receptors

**Fig. 3:**
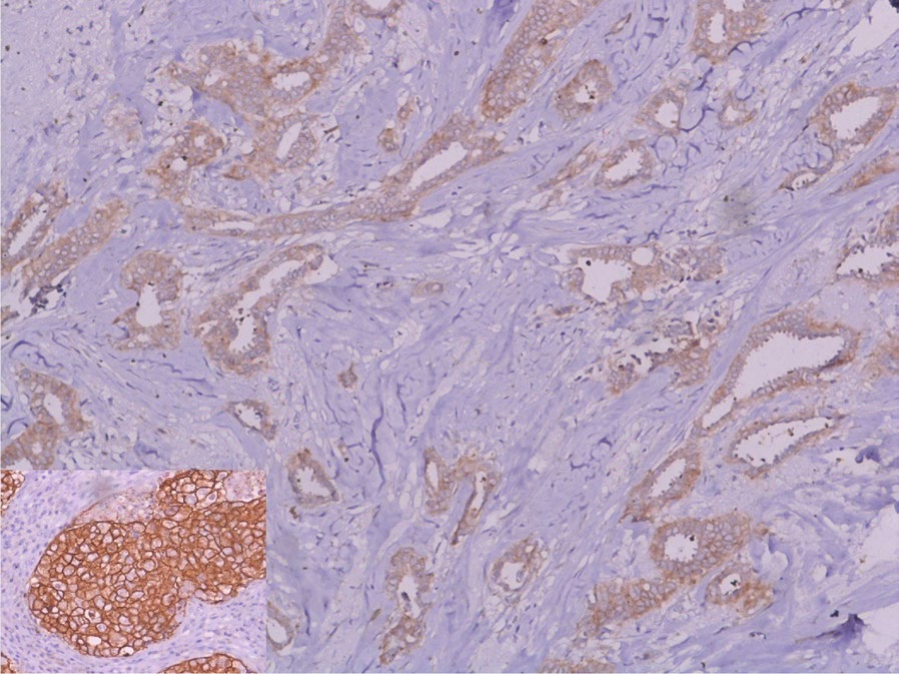
IHC × 100: *HER2* showing negative staining with positive external control at the bottom left

**Fig. 4:**
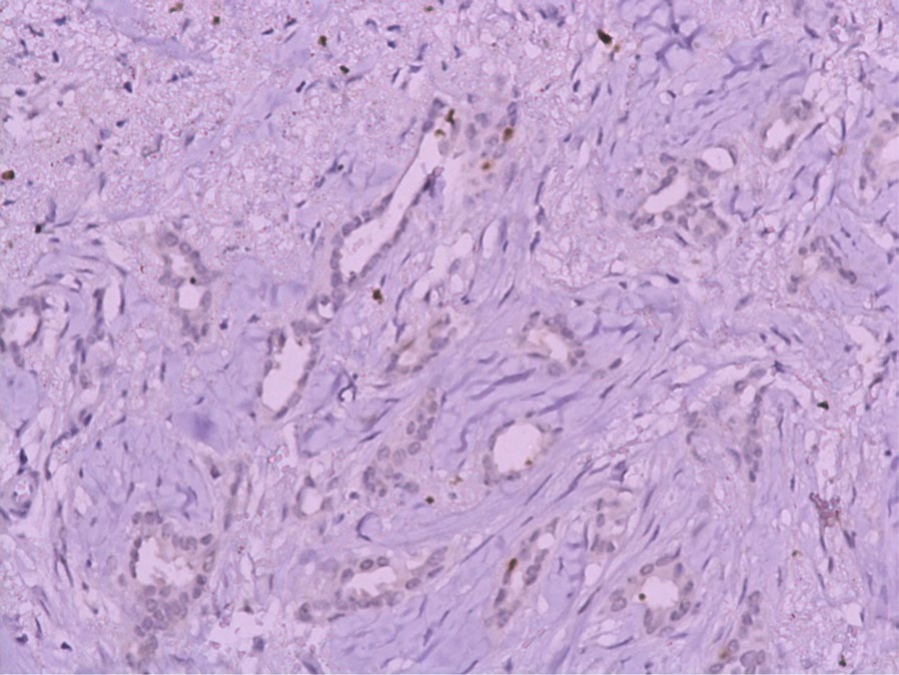
IHC for Ki67 showing a low Ki67 proliferation index (5%)

PTC tends to have a low grade. On Immunohistochemistry (IHC) analysis, this type of carcinoma is always positive for estrogen (ER) and progesterone receptors (PR), has a low growth fraction, and is typically negative for *HER2* (Figs. 2, 3). Cases with a high grade, the negativity of ER or PR, or the positivity of *HER2* should be considered as an erroneous diagnosis [9, 10]. The same results were found in our study: all patients were both ER and PR positive and negative for *HER2*/neu. Only one patient had a profile luminal B, and the others patients had a profile luminal A (Fig. 4).

Because of the small size of the tumor, the majority of patients underwent breast-conserving surgery.

The use of adjuvant therapy after breast-conserving surgery is not automatically offered. In fact, some have challenged the need for radiotherapy and adjuvant systemic therapy. Some authors recommended the use of radiotherapy after breast-conserving surgery and, in the case of metastatic axillary lymph nodes, to reduce recurrence. Hence, the main parameter to indicate adjuvant therapy is axillary macrometastasis [11].

In the current study, adjuvant radiotherapy was indicated after conservative surgery, and only in one case of radical mastectomy. Chemotherapy was indicated in the case of positive axillary lymph nodes.

Fritz *et al.* found that postoperative radiotherapy was associated with better survival. In fact, the 10-year survival rate in the radiotherapy group was 85.9% compared with 76.3% in the no radiotherapy group (*p* = 0.035). Also, they demonstrated that radiotherapy has a significant impact on relapse-free survival (100% five-year relapse-free survival with radiotherapy and 89% without radiotherapy) [11].

Similarly, Xian Chen *et al.* found that postoperative radiotherapy gives better 10-year disease-free survival (DFS) and OS (overall survival) in patients aged < 50 years, but no significant absolute survival [9].

On the other hand, other authors think that since PTC has an excellent prognosis, the role of radiotherapy is still controversial. Hamadeo *et al.* found that adjuvant radiotherapy after breast conservation has a benefit to reduce local recurrence (0% versus 5%) [8, 10].

In our study, we did not find any recurrence, even in the case of radiotherapy omission, in conservative surgery.

Because PTC is usually diagnosed during screening mammography, stage T3 or T4 are rarely observed. Authors individualized the criteria to indicate chemotherapy that is based on tumor size, grade, and lymph node status. But the current study found that chemotherapy is typically not recommended, due to the excellent prognosis, low risk for recurrence, and adverse side effects, and this subtype of carcinoma appears to have no benefit from chemotherapy [10].

In the majority of cases, PTC expresses hormone receptors. Hence, adjuvant hormonal treatment is applied to most patients because endocrine therapy diminishes the small risk of disease recurrence [11].

However, some authors, such as Fritz *et al.*, demonstrate that the use of antihormonal treatment showed no survival benefit (coefficient 1.21, *z* = 2.23, *p* = 0,026) [7].

In the present study, all patients with hormone-positive cancers were offered endocrine treatment (tamoxifen or aromatase inhibitor).

Currently, the 2015 National Comprehensive Cancer Network guidelines do not recommend adjuvant endocrine therapy for patients with tumors less than 10 mm and favorable histology, such as PTC with or without micrometastasis or isolated tumor cells in lymph nodes.

In conclusion, because of the excellent prognosis of PTC observed in the literature, even in patients who did not receive adjuvant therapy, patients should be implicated in the choice of this treatment [7].

As demonstrated, PTC is associated with a low incidence of lymph node metastases (approximately 2–11%), a low incidence of local recurrence (8% or less after mastectomy), and excellent overall survival. It behaves like other low-grade luminal A-type breast carcinomas [7]. The 5-year disease-free survival (DFS) rate is generally more than 90%, and the 10-year overall survival (OS) rate is comparable to that of the general population [4, 7].

## Conclusion

PTC is rare, it has distinct biology and clinical features, and it is usually diagnosed incidentally in screening mammography. Typically, it is characterized by a small-size, steroid receptor-positive, and c-erb-B2 negative tumor, without evidence of L1 and V1, and very few cases of lymph node metastasis. The treatment is based on surgery. The role of adjuvant therapy is controversial. The prognosis is excellent.

## Data Availability

Data supporting our findings were taken from the patient’s folder.
